# Epidemiology and Pathogenicity Analysis Based on Partial Recombinant PRRSV Strains in China

**DOI:** 10.1155/tbed/1748117

**Published:** 2025-08-13

**Authors:** Shangshang You, Lingguang Li, Junna Wang, Lingzhi Luo, Junhan Song, Houbin Ju, Lingxue Yu, Guoxin Li, Jin Cui

**Affiliations:** ^1^College of Veterinary Medicine, Northeast Agricultural University, Harbin, China; ^2^Shanghai Veterinary Research Institute, Chinese Academy of Agricultural Sciences, Shanghai, China; ^3^Veterinary Disease Diagnostic Center, Shanghai Animal Disease Control Center, Shanghai, China; ^4^Jiangsu Co-innovation Center for Prevention and Control of Important Animal Infectious Diseases and Zoonoses, Yangzhou University, Yangzhou, China

**Keywords:** genetic evolution, genetic recombination, pathogenicity, PRRSV

## Abstract

Currently, multiple recombinant variants of porcine reproductive and respiratory syndrome virus (PRRSV) are circulating in China. These variants exhibit differences in pathogenicity. To comprehensively understand the current status of the virus and its recombination patterns, a total of 677 PRRSV strains were used for evolutionary analysis, of which 673 strains were isolated from mainland China. The results indicated that current PRRSV strains in circulation in China are highly complex, with NADC30-like strains having replaced highly pathogenic strain of PRRSV (HP-PRRSV) strains as the dominant strains. An analysis of the patterns and hotspots of PRRSV-2 recombinant strains from 2019 to 2023 revealed two main types: the strains with NADC30 as the primary parent were observed to have recombination hotspots concentrated in the Nsp1, Nsp4 to Nsp9, and ORF2 to ORF6 regions, while recombination frequencies were found to be lower in the Nsp2, Nsp10, and Nsp11 regions. In contrast, the other type of recombinant strain, primarily derived from HP-PRRSV, exhibited recombination hotspots concentrated in the Nsp2 and ORF2 to ORF6 regions, while the recombination frequency in the Nsp8 and Nsp9 regions was relatively low. Further, a comprehensive analysis of the phylogenetic and recombination patterns of four PRRSV strains (ZJ-2021-1, JS-2020-1, GZ-2022-1, and SH-2020-2) indicated variations in genotyping precision among Nsp2, ORF5, and the entire genome. As demonstrated by the results of the animal experiments, there were differences in pathogenicity between ZJ-2021-1 and JS-2020-1. The pathogenicity of the recombinant strain exhibited similar characteristics to its parental skeleton. Our results provided reference data for exploring epidemiological characteristics and pathogenicity of recombinant PRRSV strains.

## 1. Introduction

Porcine reproductive and respiratory syndrome virus (PRRSV) belongs to the family *Arteriviridae*, order *Nidoviridae* [1]. PRRSV is an enveloped, single-stranded positive-sense RNA virus containing a genome of ~15 kb [2]. The PRRSV genome includes at least 10 open reading frames (ORFs) and encodes eight structural proteins and at least 16 nonstructural proteins. The nonstructural proteins are encoded as polyprotein precursors from two ORFs, ORF1a and ORF1ab. The precursors are thought to be cleaved into 16 nonstructural proteins (nsp1, nsp1, nsp2 to nsp6, nsp2N, nsp2TF, nsp7a, nsp7b, and nsp8 to nsp12) by four virus-encoded proteases upon infection [3 –5]. Based on the whole genome, PRRSV can be typically classified into two species *Betaarterivirus suid* 1 (former PRRSV-1, European type) and *Betaarterivirus suid* 2 (former PRRSV-2, American type), both of which have only about 60% homology at the nucleotide level [6, 7].

The Nsp2 and ORF5 genes demonstrate the highest variability in the PRRSV genome and are consequently extensively utilized in the analysis of PRRSV epidemiology and genetic evolution [8–10]. Previous studies indicated that PRRSV-2 strains can be categorized into nine distinct lineages based on ORF5 gene [10]. In 1996, PRRSV was first reported in mainland China [11]. In 2006, a highly pathogenic strain of PRRSV (HP-PRRSV) emerged [12]. In 2013, the NADC30-like strain was introduced and became the predominant strain in China [13]. Significantly, in 2017, China first isolated the RFLP 1-7-4 PRRSV (LNWK96 and LNWK130), from clinical specimens, and due to their high genetic similarity to the U.S. IA/2014/NADC34 strain, this lineage was designated as NADC34-like [14, 15]. PRRSV was continued to undergo mutations, and its epidemic trend was also constantly changing [16]. In recent years, reports of PRRSV recombination have increased considerably. It has been demonstrated that recombinants of PRRSV include those between circulating strains, and that those between circulating and vaccine strains may also lead to alterations in pathogenicity [17, 18]. In consideration of the evolutionary diversity of PRRSV, particularly the phenomenon of viral recombination, the prevention and control of PRRS are facing increasingly severe challenges.

In this study, we employed phylogenetic and recombination analysis of 677 PRRSV strains, including four isolated recombinant strains ZJ-2021-1, JS-2020-1, GZ-2022-1, and SH-2020-2, to evaluate the current PRRSV epidemiological status. Our study aimed to provide a reference for PRRSV epidemiological investigations, detection, and diagnosis.

## 2. Materials and Methods

### 2.1. Ethics Statements

Animal experiment was performed in accordance with the guidelines of the Institute of Shanghai veterinary research, Chinese Academy Agriculture of Sciences, using protocols approved by the Institutional Laboratory Animal Care and Use Committee (SV-20220218-01).

### 2.2. Data Set

A total of 677 complete PRRSV genome sequences were retrieved from NCBI database, including 673 Chinese isolates spanning 1996–2023 (Table S1). This dataset expanded upon the scope of our previous study [19], by incorporating an additional 312 sequences from 2018 to 2023.

### 2.3. Sequence Alignment and Phylogenetic Analysis

The sequence alignment and genetic recombination analysis were performed as described previously [19] with several modifications, such as expanded reference sequences (NADC30, NADC34, JXA1, VR2332, and QYYZ) to account for emerging lineages, and a stricter validation threshold requiring consensus between RDP4 and Simplot software (version 3.5), with a window size of 500 bp and a step size of 20 bp [20–22].

### 2.4. Clinical Samples Collection

Lung tissue and serum samples (*n* = 310) were collected from porcine abortion cases and respiratory-diseased pigs across nine Chinese provinces (e.g., Shanghai, Zhejiang, Jiangsu, and Anhui) during 2019–2022. Tissue homogenization was performed using cryogenic grinding systems (Shanghai Jingxin), followed by RNA extraction with TIANamp Viral DNA/RNA Kit (TIANGEN) according to manufacturer's protocol. For the PRRSV detection, specific primers for the amplification of the viral ORF1A, ORF5, and ORF6 gene were designed. The detailed sequence information was summarized in Table 1.

### 2.5. Genome Sequencing and Analysis

The positive PCR product was purified using E.Z.N.A. Gel Extraction Kit (Omega, China) and sequenced by Songon (Shanghai, China). Next-generation sequencing (NGS) was carried out by Shanghai Tanpu Biotechnology Co., Ltd (Shanghai, China) and the sequences of each fragment were spliced into a complete viral genome by SEQMAN software. The ORF5 and Nsp2 fragments of the virus were intercepted by MEGALIGN to analyze the whole genome and each fragment. MEGA7.0 (neighbor-joining; bootstrap values of 1000 replicates) was used to analyze the genetic evolution of the whole genome, Nsp2 and ORF5 genes of PRRSV isolated in NCBI and this study. The lineages were classified according to the genetic relationship among the genome sequences (sampled during 2019–2022) obtained in this study with 27 reference sequences (Table 2).

### 2.6. Virus Isolation

PRRSV-positive specimens identified by RT-PCR were propagated in porcine alveolar macrophage (PAM) cultured in six-well plates. Inoculated plates were incubated at 37°C with 5% CO_2_ for 2 h. Following inoculum removal, cells were supplemented with 2 mL DMEM containing 2% fetal bovine serum (FBS) and 1% streptomycin–penicillin. At 48 h postinfection, virus in supernatants were collected and storage at −80°C.

### 2.7. Indirect Immunofluorescence Assay (IFA)

At 24 h postinfection, PAMs were fixed with precooled methanol at 4°C for 15 min. After washing, cells were blocked with 5% skimmed milk. Subsequently, cells were incubated with PRRSV N-specific monoclonal antibody developed in our lab, followed by Alexa Fluor 488-conjugated donkey antimouse IgG (Jackson ImmunoResearch Laboratory, Philadelphia, PA, USA) [23] as the secondary antibody. The stained cells were visualized with a fluorescence microscope (ZEISS Axiovert 5, ZEISS, Germany).

### 2.8. Pathogenicity to Piglets

To assess the pathogenicity of the recombinant strains, nine 30-day-old healthy piglets (negative for PRRS antigen and antibody) were divided into three groups with three piglets in each group and housed separately in biosafety rooms. Two groups were inoculated with two different strains, the dose was intramuscular injection of 2 mL/pig and a nasal drop of 2 mL/pig (10^2.5^ copies/mL) and the negative control group was inoculated with an equal volume of DMEM. Following inoculation, all piglets were monitored daily for clinical symptoms and rectal temperature. On days 2, 5, 7, 10, 14, and 21 postinoculation, blood samples and nasal swabs were collected from the piglets. Serum-specific antibodies were detected using an ELISA kit (IDEXX Laboratories Inc., Westbrook, ME, USA). Viral viremia was detected by quantitative RT-qPCR assay. On day 21 postinfection, all the piglets were euthanized. During necropsy, spleen, lung, kidney, and lymph node samples were collected and subjected to quantitative RT-qPCR to determine viral load.

### 2.9. Statistical Analysis

The statistical analysis was performed using one-or two-way ANOVA followed by *t* test analysis of variance in GraphPad Prism software (version 6.0). A value of *p* less than 0.05 was considered statistically significant.

## 3. Results

### 3.1. Evolutionary Status of PRRSV in China

**Figure 1:**
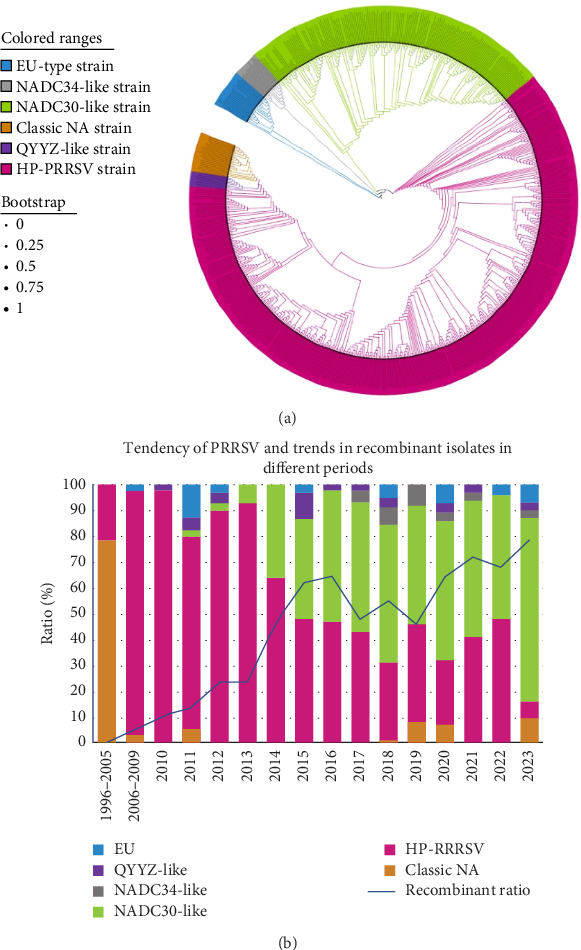
Analysis of the epidemiology of PRRSV. (A) Phylogenetic tree based on complete genome sequence analysis of PRRSV isolates. All isolates were grouped into six subgroups: EU-type strain, NADC34-like strain, NADC30-like strain, HP-PRRSV strain, QYYZ-like strain, and Classic NA strain, which are marked in blue, gray, green, pink, purple, and orange, respectively. The phylogenetic tree was generated using the NJ-tree model and assessed for reliability through 1000 bootstrap iterations. (B) Tendency of PRRSV epidemiology and recombination over time, illustrating the prevalence of recombinant isolates across different periods.

Phylogenetic analysis classified PRRSV isolates into six distinct genetic subgroups: European-type, NADC34-like, NADC30-like, HP-PRRSV, QYYZ-like, and Classic North American (NA) strains. PRRSV-2 was the predominant viral subtype (Figure 1A). An epidemiological investigation revealed that PRRSV evolved in a complex manner in China. The evolution was characterized by the cocirculation of multiple PRRSV-2 lineages, which resulted in an increase in genetic diversity. The epidemic progression exhibited four distinct phases: the initial phase (1996–2006) was characterized by relative viral stability and dominance of classical strains. This equilibrium was disrupted in 2006 with the emergence of HP-PRRSV strains with high pathogenicity. Since 2013, the subsequent phase had seen the concurrent prevalence of NADC30-like strains alongside the continued circulation of HP-PRRSV. Recently, surveillance data from 2018 to 2023 demonstrated an increase in the detection of NADC34-like strains, European-type strains, and QYYZ-like strains. This was accompanied by emerging evidence of recombinant strain formation. Collectively, these findings indicated an increasingly intricate epidemiological landscape (Figure 1B).

### 3.2. PRRSV Recombination Patterns in China

Molecular epidemiological surveillance revealed temporal dynamics in PRRSV recombination patterns. No recombinant strains were identified prior to 2006. During the initial phase of viral evolution 2006–2011, the recombination frequency remained below 10%. The subsequent period 2012–2013 saw a gradual increase in genetic recombination events, stabilizing at a prevalence of 20%–30%. Post-2014 surveillance data indicated a substantial rise in recombination rates, exceeding a detection frequency of 50% (Figure 1B). No recombinant strains were detected in the EU-type, NADC34-like, or Classic NA group. However, recombinant strains frequently occurred in the NADC30-like and HP-PRRSV from 2014 to 2023.

**Figure 2:**
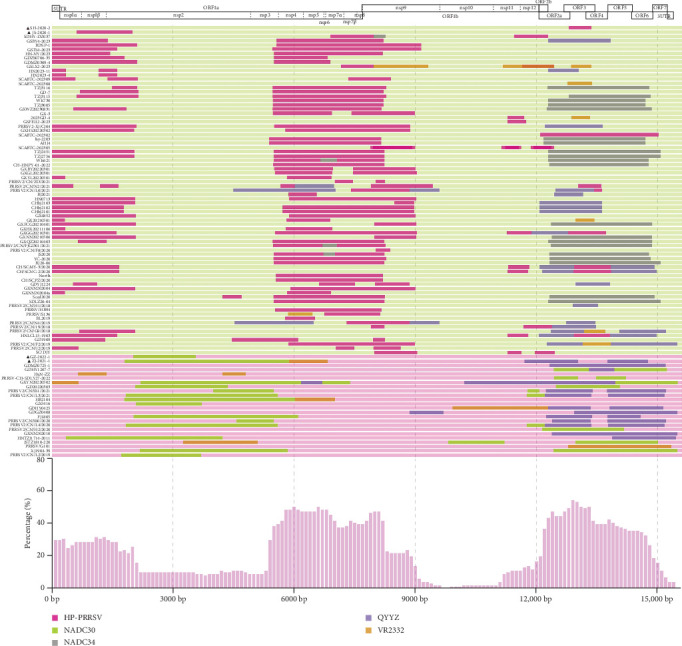
Map of parental lineages of genomes and inter-lineage recombination patterns in Chinese NA-type PRRSVs from 2019 to 2023.The top of the figure was the full-length genome structure, referenced to the VR-2332 strain, showing the positions and boundaries of the major ORFs in ORF1a and ORF1b. Distinct colors denote separate PRRSV lineages.

To explore variation in recombination among PRRSV branches in China during this period, we calculated the proportion of different recombination patterns. The most frequent interlineage recombination pattern was sublineage 1.8 (major parent) + sublineage 8.7 (minor parent), followed by sublineage 8.7 (major parent) + sublineage 1.8 (minor parent). Additionally, the proportion of recombinants based on the sublineage 1.8 backbone remained at 70% from 2019 to 2023, while the proportion based on the sublineage 8.7 backbone remained at 30%. Recombination events became increasingly complex from 2019 to 2023, with triple recombinants constituting 50.50%. The most significant pattern was characterized by sublineage 1.8 (major parent) + sublineage 8.7 (minor parent) + sublineage 1.5 (minor parent). Additionally, quadruple recombinants were partially detected, with the main recombination pattern featuring sublineage 1.8 (major parent) + L3 (minor parent) + L5 (minor parent) + sublineage 8.7 (minor parent). In summary, the proportion of recombinant strains derived from NADC30 as the major strain was currently higher than that derived from HP-PRRSV in China, and the recombination patterns were relatively complex (Figure 2). Based on the results of the recombination analysis (Figure 2), it was anticipated that recombinant strains from NADC30 as the major parent exhibit recombination hotspots in Nsp1, Nsp4 to Nsp9, and ORF2 to ORF6, while lower frequencies are observed in Nsp2, Nsp10, and Nsp11. Conversely, recombinant strains from HP-PRRSV as the major parent exhibit recombination hotspots in Nsp2 and ORF2 to ORF6, with lower frequencies are observed in Nsp8 and Nsp9.

### 3.3. Phylogenetic Analysis of PRRSV From Clinical Samples

Real-time RT-PCR results indicated that out of the 310 blood and lung samples analyzed, 115/310 (37.0%) tested positive solely for PRRSV-2, with individual farm positive rates ranging from 28.0% to 100%. Notably, PRRSV-1 was not detected in any of the samples. Four representative samples were selected from the 115 positive specimens for virus isolation on PAM and whole genome sequencing. By analyzing the samples and comparing the genomic sequencing of Nsp2, ORF5 and whole-genome, the four samples designated as ZJ-2021-1, JS-2020-1, GZ-2022-1, and SH-2020-2.

**Figure 3:**
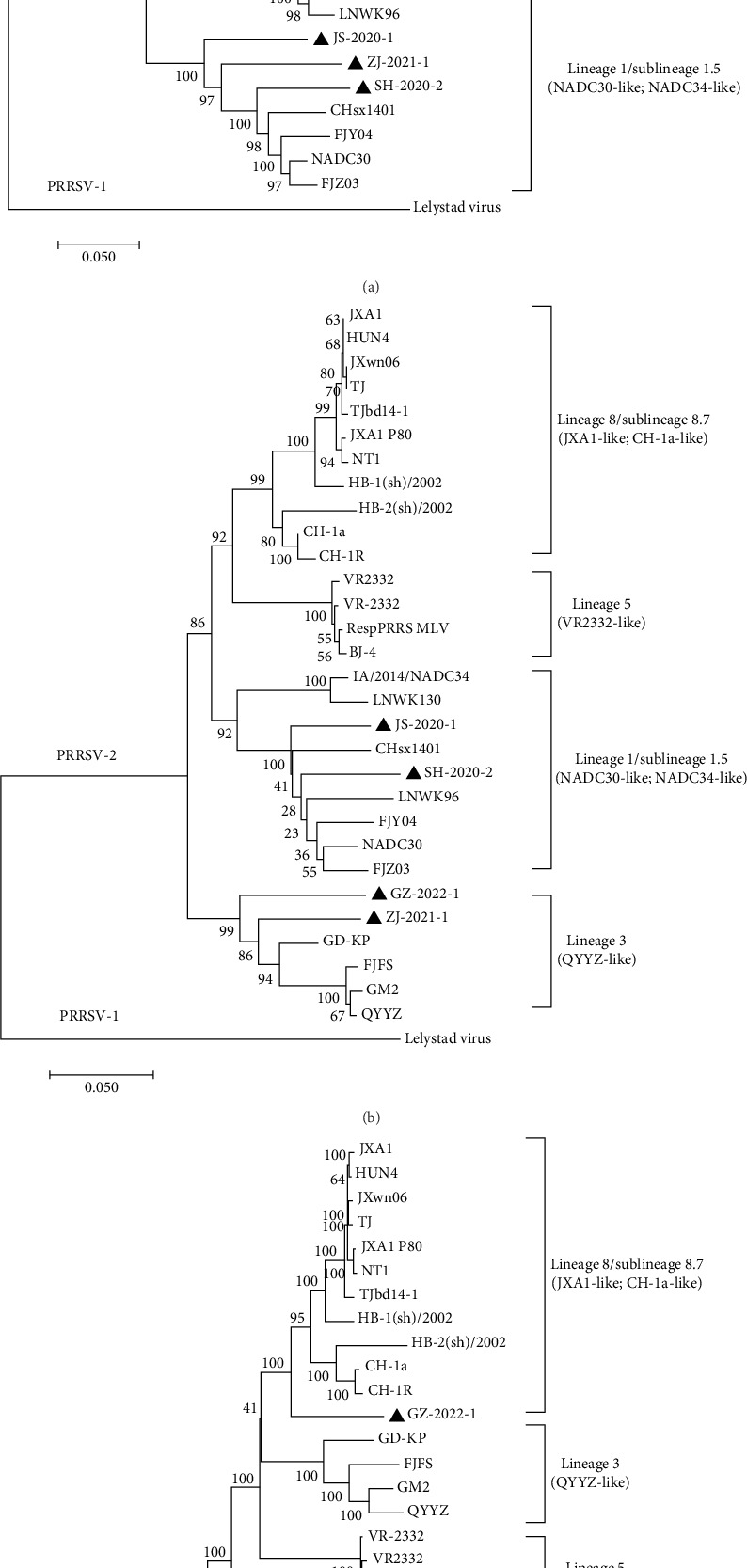
Sequence alignment and phylogenetic analysis of four isolated PRRSV strains. Phylogenetic trees were constructed using the (A) Nsp2, (B) ORF5, and (C) full-length genome. The four PRRSV strains isolated in this study were marked with black triangles.

Three phylogenetic trees were constructed based on the Nsp2, ORF5, and whole-genome nucleotide sequences of four PRRSVs isolates and 27 representative strains. The tree based on the Nsp2 sequence showed that both of them belonged to the lineage 1 branch expect GZ-2022-1. According to the phylogenetic tree of ORF5, ZJ-2021-1 and GZ-2022-1 belonged to lineage 3 and JS-2020-1 and SH-2020-2 belonged to lineage 1. Interestingly, the phylogenetic tree based on the whole-genome sequence showed that JS-2020-1 and SH-2020-2 belonged to lineage 1, while ZJ-2021-1 and GZ-2022-1 belonged to a separate lineage and were also related to other lineages (Figure 3).

### 3.4. Different Recombination Events Revealed in the Four Strains

**Figure 4:**
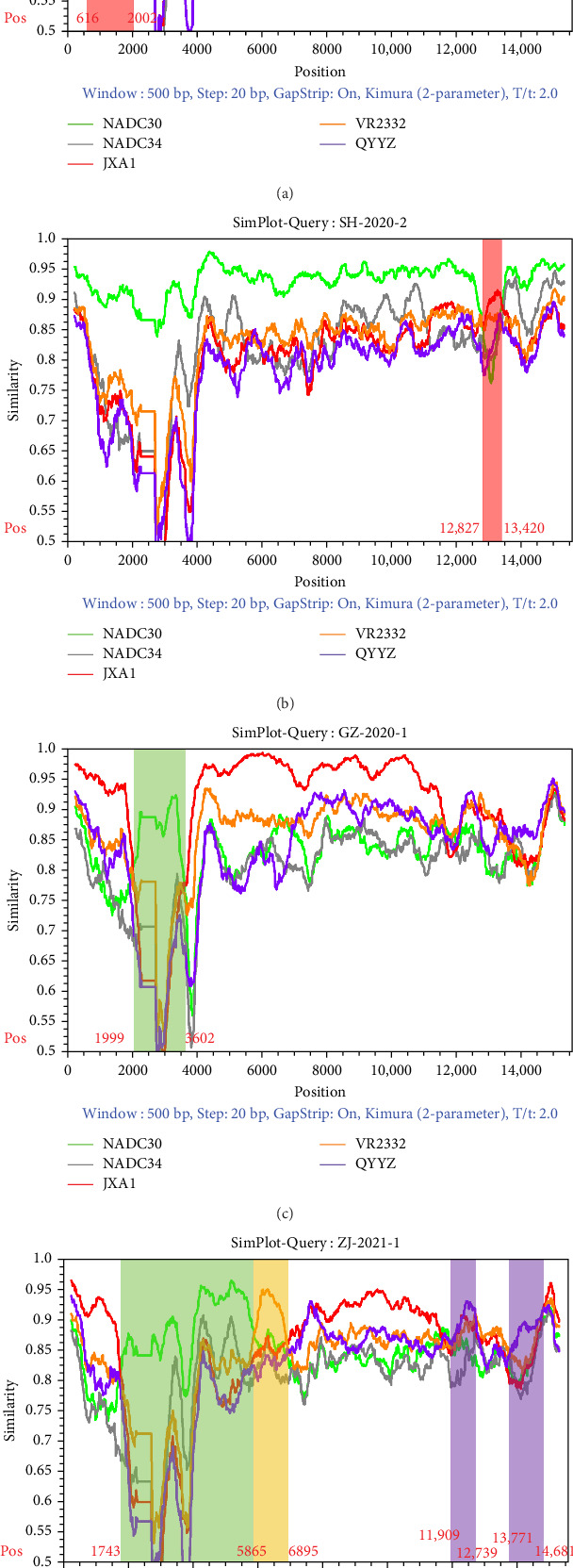
Recombination analysis of JS-2020-1 (A), SH-2020-2 (B), GZ-2022-1(C), and ZJ-2021-1 (D). The parent strains were NADC30 (green), NADC34 (gray), JXA1 (red), VR2332 (orange), and QYYZ (purple). For similarity plot analysis, the window was set size to 500 bp and the step was set size to 20 bp.

Recombination signal was detected for all four strains in RDP4 (Table S2). NADC30 was the major parent of the JS-2020-1 and SH-2020-2 strain, and JXA1 was the major parent of the ZJ-2021-1 and GZ-2022-1 strain. Simplot analysis was conducted to validate the results from RDP4. The results identified that two breakpoints were identified within JS-2020-1 and SH-2020-2, which one was located in Nsp1 (nt 616 and nt 2002), other was located in GP4 (nt 12,827 and nt 13,420). A recombination analysis revealed two potential recombination break-points at nucleotide positions Nsp2 (nt 1999 and nt 3602) in GZ-2022-1, and seven breakpoints were identified in ZJ-2021-1; one was located in the Nsp2 (nt 1743) and the remaining break-points were located in Nsp3 (nt 5865), Nsp5 (nt 6895), GP2 (nt 11,909), GP3 (nt 12,739), GP4 (nt 13,771), and GP5 (nt 14,681) (Figure 4).

### 3.5. ZJ-2021-1 and JS-2020-1 Pathogenicity for Pigs

**Figure 5:**
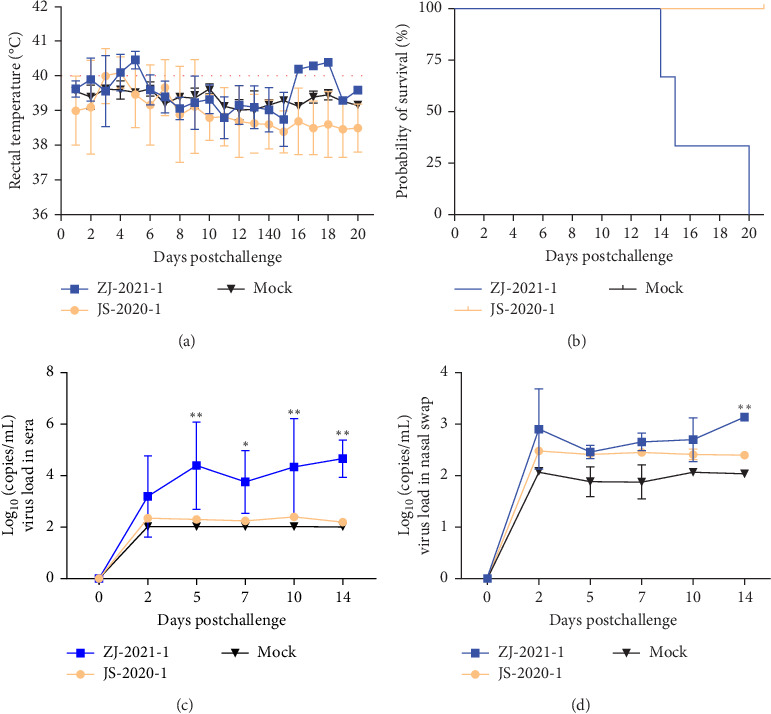
The clinical signs of piglets in the inoculated and control groups. (A) Rectal temperature. Daily rectal temperature measurements were taken, with a temperature of ≥40.0°C indicating a fever. (B) The survival and mortality curves of the inoculated piglets. (C) The amount of virus in sera and (D) nasal swabs (ns, *p*  > 0.05; *⁣*^*∗*^, *p*  < 0.05;  ^*∗*^^*∗*^, *p*  < 0.01;  ^*∗*^^*∗*^, *p*  < 0.001).

After inoculation, the inoculated piglets exhibited a significant rise in body temperature, beginning on day 1. The JS-2020-1 group began to drop in body temperature after 5 days postinoculation (dpi) and continued until the end of the experiment. Clinical signs of the piglets challenged with the ZJ-2021-1 strain included a persistent fever (>40°C) from 2 to 5 dpi, with a peak of 40.7°C at 5 dpi (Figure 5A). In contrast, the piglets in the negative control group showed no abnormal temperature fluctuations throughout the experiment period. (Figure 5A).

Pigs infected with the ZJ-2021-1 strain displayed distinct clinical signs, such as respiratory distress, anorexia, and coughing. All ZJ-2021-1-infected pigs died at 14, 15, and 20 dpi, whereas no mortality was observed in JS-2020-1-infected group and negative control group (Figure 5B).

To evaluate the vivo viral replication capacity of the experimental strain, viremia levels in piglets were measured. The analysis revealed that serum viral levels increased rapidly in the ZJ-2021-1 group and reached a peak at 5 dpi (2.51 × 10^4^ copies/mL), significantly higher (*p* < 0.01) than those in the JS-2020-1-inoculated group (Figure 5C).

Regarding the virus content in the nasal secretions of the infected group, the virus content in the ZJ-2021-1 group continued to increase and reached the peak at 14 dpi (1.34 × 10^3^ copies/mL), significantly lower (*p* < 0.01) copy number virus content (2.51 × 10^2^ copies/mL) was detected in the JS-2020-1 group, while viremia and excretion in the blank control group were consistently in the negative range throughout the study (Figure 5D).

**Figure 6:**
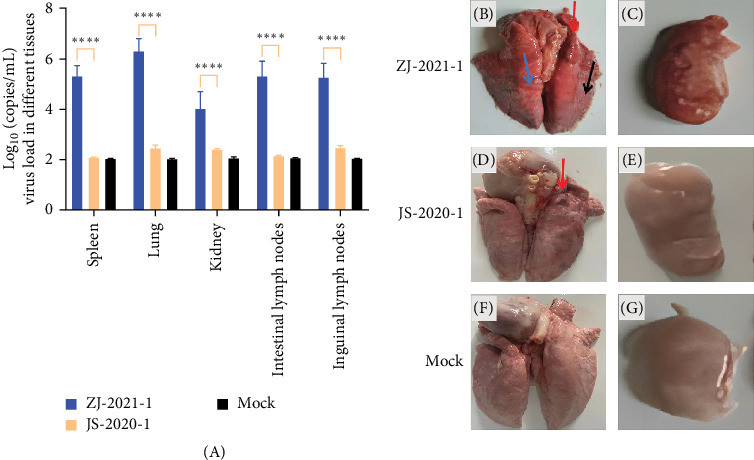
The pathological changes observed in the piglets' lungs and the viral load testing results after inoculation with the ZJ-2021-1 and JS-2020-1 strains. (A) Results of viral load in piglets from different groups after challenge. (B–C) Lung autopsy pictures from the ZJ-2021-1 group. (D–E) Lung autopsy pictures from the JS-2020-1 group. (F–G) Lung autopsy pictures from the control group (ns, *p*  > 0.05; *⁣*^*∗*^, *p*  < 0.05;  ^*∗*^^*∗*^, *p*  < 0.01;  ^*∗*^^*∗*^, *p*  < 0.001).

Additionally, samples were collected from the spleen, lungs, kidneys, intestinal lymph nodes, and inguinal lymph nodes to determine the viral load in these organs. The results revealed that the lungs exhibited the highest viral load (1.9 × 10^6^ copies/mL), while the kidney of the ZJ-2021-1 group exhibited the lowest viral load (1× 10^4^ copies/mL). In contrast, significantly lower (*p* < 0.001) levels of viral load were detected in the JS-2020-1 group (Figure 6A). On the 14th day of the experiment, the piglets were euthanized for observation. Analysis of ocular lesions in lung tissues revealed normal lung tissue in piglets in the JS-2020-1 and control groups, while significant lung lesions were evident in the ZJ-2021-1 inoculated group (Figure 6B). In Figure 6, the red, blue, and black arrows indicate fleshy lung lesions, diffuse hemorrhagic spots, and bruised lung lesions, respectively. The inguinal lymph nodes in the ZJ-2021-1 group exhibited bleeding symptoms.

## 4. Discussion

PRRS stands out as one of the most economically significant diseases in the pig industry and has been persistently prevalent across China for several decades [24]. There are classical strains, HP-PRRSV strains, NADC30-like strains, and NADC34-like strains circulating in China [25–27]. In recent years, European strains had also been detected, which made it more difficult to detect and control PRRSV [28, 29]. Gene recombination was one of the ways of genetic variation and evolution of viruses [30]. In this study, we analyzed the genetic recombination characteristics of PRRSV-2 from 2019 to 2023, and the results revealed a complex trend in viral genome recombination. In terms of recombination patterns, the combination of sublineage 1.8 (major parent) and sublineage 8.7 was the most common. Notably, the proportion of samples with triple-recombinants reached 50.50%, which was similar to the 62.50% recombination rate reported by Zhang et al. [18]. From an epidemiological perspective, the dominance of sublineage 1.8 in recombination events may be related to its strong adaptability and high transmission efficiency. The recombination phenomena between sublineage 1.8 and others, such as sublineage 8.7 and sublineage 1.5, may represent an important evolutionary mechanism for the virus to cope with host immune pressure and environmental selection [24]. Epidemiological data indicates that the prevalence of NADC30 has been steadily increasing since 2016. By 2020, it has become the predominant strain, surpassing HP-PRRSV and creating favorable conditions for viral recombination [26]. In summary, the proportion of recombinant strains derived from NADC30 as the major strain was currently higher than that derived from HP-PRRSV in China, and the recombination patterns were relatively complex.

The complex recombination of PRRSV increased its genetic diversity, posing potential risks to the efficacy, and safety of vaccines [31]. Since the introduction of NADC30-like strains into China in 2013, there has been a growing number of reports documenting recombination events involving vaccine-derived strains of PRRSV [32, 33]. For example, FJW05, FJXS15, 15JX1, 15HEN1, and 15SC3 were all recombinant viruses derived from the attenuated vaccine strains JXA1-R and NADC30-like strains [34, 35]. The high frequency of genetic recombination and the relatively weak immunogenicity of current vaccine candidates had significantly limited their protective efficacy. Moreover, hyperimmune sera derived from existing vaccine strains wereunable to effectively neutralize certain recombinant strains, such as FJLIUY2017 and PRRSV2/CN/G8/2018 [36]. We attempted to analyze the impact of PRRSV recombination on vaccine protection efficacy. However, owing to the high variability and complex recombination mechanisms of PRRSV, current research is still in the exploratory stage. The ORF2-6 region in PRRSV has been confirmed to be associated with viral virulence and immune evasion, and changes in these regions may weaken the protective efficacy of existing vaccines [37]. Based on our analysis of PRRSV-2 recombination patterns and hotspot predictions from 2019 to 2023, there were two main types of recombinant strains in China: those with NADC30 as the major parent, which showed recombination hotspots in Nsp1, Nsp4 to Nsp9, and ORF2 to ORF6, and had lower frequencies in Nsp2, Nsp10, and Nsp11; and those with HP-PRRSV as the major parent, which had hotspots in Nsp2 and ORF2 to ORF6, and had lower frequencies in Nsp8 and Nsp9. By systematically dissecting the structure and function of these recombination hotspots, we can identify highly conserved epitopes or functional domains that could serve as key targets for diagnostic targets. These findings have significant implications for understanding the evolutionary dynamics and genetic diversity of PRRSV and provide a theoretical foundation for developing novel diagnostic methods. For instance, we can (i) develop multiplex PCR detection methods targeting recombination hotspot regions; (ii) create rapid test strips based on sequence variations in these hotspot regions; and (iii) performe virus tracing and evolutionary studies utilizing the results of recombination analysis. By establishing and implementing these methodologies, it became feasible to effectively monitor and diagnosed PRRSV recombinant strains, thereby providing a scientific foundation for PRRSV control. Furthermore, these approached offer novel insights and tools for detecting recombinant strains of other viruses.

Traditional PRRSV subtyping had predominantly relied on the highly variable ORF5; however, given that the ORF5 sequence constitutes merely 5% of the entire PRRSV genome, classification predicated solely on ORF5 failed to encompass the entire gamut of genetic variations among PRRSV strains and the considerable mutability of Nsp2 may impose complications on sequencing and analytical endeavors [38]. For instance, in the case of ZJ-2021-1, Nsp2 sequence analysis indicated it belongs to lineage 1, while traditional ORF5 analysis placed it in lineage 3; underscoring the risk of misclassification when relying solely on ORF5. Based on our analysis of PRRSV-2 recombination patterns and hotspot predictions from 2019 to 2023, ORF5 had been identified as a frequent recombination site, so it is not representative for genotyping recombinant strains. A case study demonstrated that whole-genome sequencing (WGS) offers greater insights into PRRSV diversity and evolution compared to ORF5-based subtyping. WGS captures the complete genetic diversity of PRRSV strains, encompassing analyses of both ORF5 and Nsp2 [39]. Therefore, integrating WGS into surveillance protocols is critical for accurate epidemiological tracking, vaccine development, and outbreak management.

Some studies had shown that the locations of NSP3-8 and ORF5 were the main virulence determinants [40]. The deletion of 30 discontinuous amino acids in the HP-PRRSV Nsp2 coding region was not related to increased virulence [41]. Nsp9 and NSp10 were the virulence determinants of HP-PRRSV [42]. A comparative analysis of the recombination features of JS-2020-1 and ZJ-2021-1 revealed that JS-2020-1 had a backbone of NADC30 and recombines with partial regions of JXA1 (nt 616 and nt 2002, located in Nsp1-Nsp2). In contrast, ZJ-2021-1 had a backbone of JXA1 and more complex recombination regions, but no recombination occurred at the positions of NSP9 and NSP10.The viral load in infected pigs was closely related to virulence. In piglets infected with ZJ-2021-1, the number of viral RNA copies in the serum was significantly greater than that in piglets infected with JS-2020-1. The viral load in various tissues (such as the spleen, lungs, kidneys, intestinal lymph nodes, and inguinal lymph nodes) was also significantly greater in the ZJ-2021-1-infected group than in the JS-2020-1-infected group.

## 5. Conclusion

In summary, the analysis of the epidemiological characteristics of recombinant PRRSV strains in China indicates that the recombination of current PRRSV strains is becoming increasingly complex, including the emergence of triploid and tetraploid recombinant strains. Integrating multiple genomic regions (including Nsp2, ORF5, and the entire genome) can improve the accuracy of PRRSV genotyping. Additionally, the pathogenicity of recombinant strains exhibits similar virulence characteristics to their viral parental backbone. Our study not only deepens our understanding of the evolutionary characteristics of recombinant PRRSV in China but also provides important information for the prevention and control of PRRS.

## Figures and Tables

**Table 1:** Primes for PRRSV detection.

Primers	Sequences(5'–3')
ORF1a-F	CACGTGTACCCCCAATGC
ORF1a-R	CCCGGCCACGTACATGAC
ORF5-F	TTGTGGTGTATCGTGCCGTTCT
ORF5-R	CGACCCCATTGTTCCGCTGAAA
ORF6-F	CACAGCTCCACAGAAGGTGC
ORF6-R	TAACAGCTTTTCTGCCACCC

**Table 2:** Representative PRRSV strains used in this study.

No.	Name	GenBank accession no.	Origin
1	BJ-4	AF331831	China
2	CH-1a	AY032626	China
3	CH-1R	EU807840	China
4	CHsx1401	KP861625	China
5	FJFS	KP998476	China
6	FJY04	KP860910	China
7	FJZ03	KP860909	China
8	GD-KP	KU978619	China
9	GM2	JN662424	China
10	HB-1(sh)/2002	AY150312	China
11	HB-2(sh)/2002	AY262352	China
12	HUN4	EF635006	China
13	JXA1 P80	FJ548855	China
14	JXA1	EF112445	China
15	JXwn06	EF641008	China
16	LV	M96262	Netherlands
17	LNWK96	MG860516	China
18	LNWK130	MG913987	China
19	NADC30	JN654459	USA
20	NADC34	MF326985	USA
21	NT1	KP179402	China
22	QYYZ	JQ308798	China
23	RespPRRS MLV	AF066183	USA
24	TJ	EU860248	China
25	TJbd14-1	KP742986	China
26	VR2332	EF536003	USA
27	VR-2332	AY150564	USA

## Data Availability

The data that support the findings of this study are available from the corresponding author upon reasonable request.

## Supporting Information

Supporting Information 1 Table S1: Information of the PRRSV reference strains used for phylogenetic analysis in this study.

Supporting Information 2 Table S2: Information on recombination events of four strains, detected by RPD4 software.
