# Supplementary material for: Epidemiology and Pathogenicity Analysis Based on Partial Recombinant PRRSV Strains in China
Source: Transbound Emerg Dis. 2025 Aug 13;2025:1748117. doi: 10.1155/tbed/1748117 (PMC12367394; doi:10.1155/tbed/1748117)
Supplement: Supporting Information 1 — Table S1: Information of the PRRSV reference strains used for phylogenetic analysis in this study. [file 1748117.f1.docx]

| Table S1 Information of the PRRSV reference strains used for phylogenetic analysis in this study. | | | | |  |
| --- | --- | --- | --- | --- | --- |
|  | Strain name | Accession number | Isolate/collection date | Country |  |
|  |  |  |  |  |  |
| 1 | Lelystadvirus(LV) | M96262 | 1991 | Netherlands |  |
| 2 | VR2332 | EF536003 | 1992 | USA |  |
| 3 | NADC30 | JN654459 | 2008 | USA |  |
| 4 | IA/2014/NADC34 | MF326985 | 2014 | USA |  |
| 5 | 15HEN1_EU | KX967492 | 2015 | China |  |
| 6 | LNEU12 | KM196101 | 2012 | China |  |
| 7 | GZ11-G1 | KF001144 | 2011 | China |  |
| 8 | BJEU06-1 | GU047344 | 2006 | China |  |
| 9 | NVDC-NM1-2011 | JX187609 | 2011 | China |  |
| 10 | NMEU09-1 | GU047345 | 2009 | China |  |
| 11 | NVDC-NM2 | KC492504 | 2011 | China |  |
| 12 | NVDC-FJ | KC492506 | 2011 | China |  |
| 13 | NVDC-NM3 | KC492505 | 2011 | China |  |
| 14 | HB-2(sh)/2002 | AY262352 | 2002 | China |  |
| 15 | HH08 | JX679179 | 2008 | China |  |
| 16 | CH-1a | AY032626 | 1996 | China |  |
| 17 | CH2004 | EU880439 | 2004 | China |  |
| 18 | CH2002 | EU880438 | 2002 | China |  |
| 19 | CH2003 | EU880440 | 2003 | China |  |
| 20 | GZ1101 | KF771273 | 2011 | China |  |
| 21 | HN1 | AY457635 | 2003 | China |  |
| 22 | PRRSV02 | FJ175688 | 2002 | China |  |
| 23 | PRRSV03 | FJ175689 | 2003 | China |  |
| 24 | DY | JN864948 | 2007 | China |  |
| 25 | YN-2011 | JX857698 | 2011 | China |  |
| 26 | CC-1 | EF153486 | 2006 | China |  |
| 27 | Clone20 | FJ899592 | 2003 | China |  |
| 28 | SD1-100 | GQ914997 | 2009 | China |  |
| 29 | PRRSV01 | FJ175687 | 2001 | China |  |
| 30 | S1 | DQ459471 | 1998 | China |  |
| 31 | BJ-4 | AF331831 | 1996 | China |  |
| 32 | GS2004 | EU880443 | 2004 | China |  |
| 33 | GS2002 | EU880441 | 2002 | China |  |
| 34 | GS2003 | EU880442 | 2003 | China |  |
| 35 | Em2007 | EU262603 | 2007 | China |  |
| 36 | GD-KP | KU978619 | 2015 | China |  |
| 37 | GM2 | JN662424 | 2011 | China |  |
| 38 | QY2010 | JQ743666 | 2010 | China |  |
| 39 | QYYZ | JQ308798 | 2011 | China |  |
| 40 | ZJXS1412 | MF669722 | 2014 | China |  |
| 41 | HeN1401 | MF766471 | 2014 | China |  |
| 42 | HeN1601 | MF766474 | 2016 | China |  |
| 43 | SDhz1512 | KX980392 | 2015 | China |  |
| 44 | SDlz1601 | KX980393 | 2016 | China |  |
| 45 | HZ-31 | KC445138 | 2012 | China |  |
| 46 | GDZS2016 | MH046843 | 2016 | China |  |
| 47 | SCya17 | MH324400 | 2017 | China |  |
| 48 | GDQYQC2 | MF526896 | 2016 | China |  |
| 49 | GDYDZZZ | KY745901 | 2016 | China |  |
| 50 | HZL1501 | MF669721 | 2015 | China |  |
| 51 | XJzx1-2015 | KX689233 | 2015 | China |  |
| 52 | SH1211 | KF678434 | 2012 | China |  |
| 53 | HLJB1 | KT351740 | 2013 | China |  |
| 54 | NJ-1106 | JX880029 | 2011 | China |  |
| 55 | NT0801 | HQ315836 | 2008 | China |  |
| 56 | BJ0706 | GQ351601 | 2007 | China |  |
| 57 | GD3 | GU269541 | 2005 | China |  |
| 58 | NB/04 | FJ536165 | 2004 | China |  |
| 59 | YN-1 | KJ747052 | 2011 | China |  |
| 60 | SHB | EU864232 | 2005 | China |  |
| 61 | HB-1(sh)/2002 | AY150312 | 2002 | China |  |
| 62 | LN1101 | KF751238 | 2011 | China |  |
| 63 | NVDC-BJ2-2011 | KP771748 | 2011 | China |  |
| 64 | ZCYZ | JF800911 | 2009 | China |  |
| 65 | NVDC-GD2-2011 | JQ715697 | 2011 | China |  |
| 66 | HN-HW | FJ797690 | 2006 | China |  |
| 67 | HUN4 | EF635006 | 2006 | China |  |
| 68 | GDQJ | GQ374441 | 2007 | China |  |
| 69 | HN2007 | EU880437 | 2007 | China |  |
| 70 | GD2007 | EU880433 | 2007 | China |  |
| 71 | SC/NJ2016 | MF818049 | 2016 | China |  |
| 72 | WUH4 | JQ326271 | 2011 | China |  |
| 73 | JX143 | EU708726 | 2006 | China |  |
| 74 | Jiangxi-3 | EU200961 | 2007 | China |  |
| 75 | FZ06A | MF370557 | 2006 | China |  |
| 76 | HEB1 | EF112447 | 2006 | China |  |
| 77 | 09HEB | JF268679 | 2009 | China |  |
| 78 | SX-1 | GQ857656 | 2009 | China |  |
| 79 | Henan-1 | EU200962 | 2007 | China |  |
| 80 | BJ | EU825723 | 2007 | China |  |
| 81 | QH-08 | KU201579 | 2008 | China |  |
| 82 | HENZZ-8 | KU950375 | 2015 | China |  |
| 83 | NVDC-SC1-2014 | KP771739 | 2014 | China |  |
| 84 | 10-10QN | JQ663556 | 2010 | China |  |
| 85 | SCwhn14DY | KT819203 | 2014 | China |  |
| 86 | BJBLZ | FJ950745 | 2007 | China |  |
| 87 | HV | JX317648 | 2007 | China |  |
| 88 | SX2007 | EU880434 | 2007 | China |  |
| 89 | YN2008 | EU880435 | 2008 | China |  |
| 90 | GS2008 | EU880431 | 2008 | China |  |
| 91 | JSyx | EU939312 | 2006 | China |  |
| 92 | WUH1 | EU187484 | 2006 | China |  |
| 93 | GDBY1 | GQ374442 | 2008 | China |  |
| 94 | GD-HD | KP793736 | 2011 | China |  |
| 95 | 09HUN2 | JF268674 | 2009 | China |  |
| 96 | 10HN-GD | JX192632 | 2010 | China |  |
| 97 | NVDC-NM-2008 | KP771779 | 2008 | China |  |
| 98 | SY0608 | EU144079 | 2006 | China |  |
| 99 | 09HUN1 | JF268673 | 2009 | China |  |
| 100 | SCwhn09CD | JN836553 | 2009 | China |  |
| 101 | NVDC-CQ1-2011 | KP771746 | 2011 | China |  |
| 102 | NVDC-CQ3-2011 | KP771774 | 2011 | China |  |
| 103 | CBB-1-F3 | FJ889129 | 2008 | China |  |
| 104 | NVDC-CQ1-2012 | KP771747 | 2012 | China |  |
| 105 | NVDC-HeB1-2013 | KP771745 | 2013 | China |  |
| 106 | NVDC-HeB2-2013 | KP771744 | 2013 | China |  |
| 107 | GX09-16 | HM214913 | 2009 | China |  |
| 108 | CG | EU864231 | 2007 | China |  |
| 109 | GDQY2 | GU454850 | 2007 | China |  |
| 110 | GDQY1 | JN387271 | 2007 | China |  |
| 111 | TP | EU864233 | 2006 | China |  |
| 112 | YN9 | GU232738 | 2008 | China |  |
| 113 | 08SDWF | GU168569 | 2008 | China |  |
| 114 | HPBEDV | EU236259 | 2007 | China |  |
| 115 | JXA1 | EF112445 | 2006 | China |  |
| 116 | SHH | EU106888 | 2006 | China |  |
| 117 | JXwn06 | EF641008 | 2006 | China |  |
| 118 | TJ | EU860248 | 2006 | China |  |
| 119 | NVDC-HeB-2008 | KP771754 | 2008 | China |  |
| 120 | BJPG | FJ950746 | 2008 | China |  |
| 121 | LN | EU109502 | 2006 | China |  |
| 122 | NVDC-CQ-2008 | KP771755 | 2008 | China |  |
| 123 | HUB1 | EF075945 | 2006 | China |  |
| 124 | HUB2 | EF112446 | 2006 | China |  |
| 125 | SY0909 | HQ315837 | 2009 | China |  |
| 126 | AH0701 | GU461292 | 2007 | China |  |
| 127 | NVDC-CQ3-2012 | KP771775 | 2012 | China |  |
| 128 | SD-CXA2008 | GQ359108 | 2008 | China |  |
| 129 | HLJ-09 | HQ843178 | 2009 | China |  |
| 130 | SX-09 | HQ843181 | 2009 | China |  |
| 131 | HLM-09 | HQ843179 | 2009 | China |  |
| 132 | SD-09 | HQ843180 | 2009 | China |  |
| 133 | SD17 | MH404256 | 2017 | China |  |
| 134 | NVDC-HeB2-2012 | KP771772 | 2012 | China |  |
| 135 | JX | JX317649 | 2010 | China |  |
| 136 | GX09-29 | HM214914 | 2009 | China |  |
| 137 | 07QN | FJ394029 | 2007 | China |  |
| 138 | 08HuN | GU169411 | 2008 | China |  |
| 139 | GX09-32 | HM214915 | 2009 | China |  |
| 140 | BJsy06 | EU097707 | 2006 | China |  |
| 141 | NX06 | EU097706 | 2006 | China |  |
| 142 | JX2006 | EU880432 | 2006 | China |  |
| 143 | XL2008 | EU880436 | 2008 | China |  |
| 144 | Henan-A5 | KJ534540 | 2013 | China |  |
| 145 | Henan-A6 | KJ534541 | 2013 | China |  |
| 146 | Henan-A7 | KJ534542 | 2013 | China |  |
| 147 | Henan-A8 | KJ534543 | 2013 | China |  |
| 148 | 14LY01-FJ | KP780881 | 2014 | China |  |
| 149 | 15LY02-FJ | KU215417 | 2015 | China |  |
| 150 | 14LY02-FJ | KP780882 | 2014 | China |  |
| 151 | 15LY01-FJ | KU215416 | 2015 | China |  |
| 152 | HuN | EF517962 | 2006 | China |  |
| 153 | BJSY07 | HM011104 | 2007 | China |  |
| 154 | BJSY-1 | FJ950744 | 2007 | China |  |
| 155 | NVDC-BJ7-2012 | KP771758 | 2012 | China |  |
| 156 | NVDC-BJ8-2012 | KP771757 | 2012 | China |  |
| 157 | XH-GD | EU624117 | 2007 | China |  |
| 158 | 10GZ-GD | JX192633 | 2010 | China |  |
| 159 | 11GZ-GD | JX235370 | 2011 | China |  |
| 160 | 11FS11-GD | JX215551 | 2011 | China |  |
| 161 | 11FS12-GD | JX215554 | 2011 | China |  |
| 162 | CWZ-1-F3 | FJ889130 | 2008 | China |  |
| 163 | NVDC-CQ4-2012 | KP771777 | 2012 | China |  |
| 164 | NVDC-CQ2-2012 | KP771776 | 2012 | China |  |
| 165 | 07BJ | FJ393459 | 2007 | China |  |
| 166 | GD | EU825724 | 2007 | China |  |
| 167 | 17-ZJ-HZ | MF770574 | 2017 | China |  |
| 168 | GD1404 | MF669720 | 2014 | China |  |
| 169 | XJu-1 | KF815525 | 2012 | China |  |
| 170 | HeN1301 | MF766470 | 2013 | China |  |
| 171 | TJbd14-1 | KP742986 | 2014 | China |  |
| 172 | TJbd14-2 | KP742987 | 2014 | China |  |
| 173 | 07HEBTJ | FJ393458 | 2007 | China |  |
| 174 | 07NM | FJ393456 | 2007 | China |  |
| 175 | NM1 | EU860249 | 2007 | China |  |
| 176 | 07HEN | FJ393457 | 2007 | China |  |
| 177 | BJSD | FJ950747 | 2007 | China |  |
| 178 | SD16 | JX087437 | 2012 | China |  |
| 179 | TA-12-P | HQ416720 | 2008 | China |  |
| 180 | 09SD | JF268678 | 2009 | China |  |
| 181 | 09BJ | JF268676 | 2009 | China |  |
| 182 | 09HUB5 | GU168568 | 2009 | China |  |
| 183 | ZP-1 | HM016159 | 2009 | China |  |
| 184 | 09HUB1 | JF268682 | 2009 | China |  |
| 185 | 09HUB2 | JF268683 | 2009 | China |  |
| 186 | 09SC | JF268672 | 2009 | China |  |
| 187 | SX2009 | FJ895329 | 2009 | China |  |
| 188 | WUH3 | HM853673 | 2008 | China |  |
| 189 | WUH2 | EU678352 | 2008 | China |  |
| 190 | 09HUB7 | GU168567 | 2009 | China |  |
| 191 | SC2012 | KM189443 | 2012 | China |  |
| 192 | HLJHL | HM189676 | 2009 | China |  |
| 193 | 10-10JL | JQ663554 | 2010 | China |  |
| 194 | 09DB1 | JF268677 | 2009 | China |  |
| 195 | 09DB2 | JF268681 | 2009 | China |  |
| 196 | NMG2014 | KM000066 | 2014 | China |  |
| 197 | HeN1502 | MF766473 | 2015 | China |  |
| 198 | HeNan-A1 | KJ002451 | 2013 | China |  |
| 199 | HeN1201 | MF689000 | 2012 | China |  |
| 200 | SDA2 | JX878379 | 2011 | China |  |
| 201 | SDA3 | JX878380 | 2011 | China |  |
| 202 | NVDC-SD4-2014 | KP771784 | 2014 | China |  |
| 203 | Henan-A3 | KJ019330 | 2013 | China |  |
| 204 | Henan-A4 | KJ534539 | 2013 | China |  |
| 205 | Henan-A13 | KJ819935 | 2014 | China |  |
| 206 | Henan-A12 | KJ819934 | 2014 | China |  |
| 207 | MY-486 | KJ609516 | 2013 | China |  |
| 208 | HLJA1 | KT351739 | 2013 | China |  |
| 209 | HeN1501 | MF766472 | 2015 | China |  |
| 210 | HeNan-A2 | KJ002452 | 2013 | China |  |
| 211 | Henan-A14 | KJ819936 | 2014 | China |  |
| 212 | HeNan-A9 | KJ546412 | 2013 | China |  |
| 213 | MY-376 | KJ609517 | 2013 | China |  |
| 214 | JXja15 | KR149645 | 2015 | China |  |
| 215 | HEB20130008-13 | KP771753 | 2013 | China |  |
| 216 | KP | GU232735 | 2008 | China |  |
| 217 | HB2014001 | KM261784 | 2014 | China |  |
| 218 | JL-0412 | JX177644 | 2012 | China |  |
| 219 | NT1 | KP179402 | 2012 | China |  |
| 220 | NVDC-SD1-2014 | KP771738 | 2014 | China |  |
| 221 | NVDC-SDXX-2013 | KP771741 | 2013 | China |  |
| 222 | 11SH1-GD | JX235366 | 2011 | China |  |
| 223 | HENZK-1 | KU950373 | 2014 | China |  |
| 224 | NVDC-HBCZ-2013 | KP771742 | 2013 | China |  |
| 225 | NVDC-MD1-2013 | KP771751 | 2013 | China |  |
| 226 | 11SH-GD | JX235365 | 2011 | China |  |
| 227 | HNP5 | KT445876 | 2014 | China |  |
| 228 | HUN-2014 | KP330232 | 2014 | China |  |
| 229 | NVDC-BJ3-2012 | KP771762 | 2012 | China |  |
| 230 | NT2 | KP179403 | 2012 | China |  |
| 231 | HENPDS-2 | KU950370 | 2015 | China |  |
| 232 | HEB20130008-14 | KP771752 | 2013 | China |  |
| 233 | NVDC-HeB1-2011 | KP771749 | 2011 | China |  |
| 234 | NVDC-JS2-2011 | JQ715698 | 2011 | China |  |
| 235 | NVDC-MD2-2013 | KP771750 | 2013 | China |  |
| 236 | HEB-2013 | KJ591659 | 2013 | China |  |
| 237 | NT3 | KP179404 | 2012 | China |  |
| 238 | Shaanxi-2 | HQ401282 | 2007 | China |  |
| 239 | HNyc13 | KT022072 | 2013 | China |  |
| 240 | NVDC-BJPG-2013 | KP771743 | 2013 | China |  |
| 241 | YD | JF748717 | 2009 | China |  |
| 242 | GZgy15-1 | KT358728 | 2015 | China |  |
| 243 | NVDC-SD6-2014 | KP771737 | 2014 | China |  |
| 244 | 11NZ-GD | JX217036 | 2011 | China |  |
| 245 | 11XX-GD | JX235367 | 2011 | China |  |
| 246 | GX1001 | JQ955657 | 2011 | China |  |
| 247 | GX1003 | JX912249 | 2010 | China |  |
| 248 | NVDC-shh01-2014 | KP771736 | 2014 | China |  |
| 249 | NVDC-SHH02-2014 | KP771735 | 2014 | China |  |
| 250 | NVDC-BJ9-2012 | KP771756 | 2012 | China |  |
| 251 | NVDC-SD2-2012 | KP771768 | 2012 | China |  |
| 252 | NVDC-13SXJC-2014 | KP771780 | 2014 | China |  |
| 253 | NVDC-SXJC-2013 | KP771740 | 2013 | China |  |
| 254 | HNxa14 | KT022071 | 2014 | China |  |
| 255 | GX1002 | JQ955658 | 2010 | China |  |
| 256 | HB-XL | KP162169 | 2014 | China |  |
| 257 | NVDC-SD1-2012 | KP771769 | 2012 | China |  |
| 258 | NVDC-BJ4-2012 | KP771761 | 2012 | China |  |
| 259 | NVDC-BJ5-2012 | KP771760 | 2012 | China |  |
| 260 | 10BY-GD | JX192636 | 2010 | China |  |
| 261 | NVDC-HeB1-2012 | KP771773 | 2012 | China |  |
| 262 | BJ1102 | KF751237 | 2011 | China |  |
| 263 | 10FS-GD | JX192634 | 2010 | China |  |
| 264 | NVDC-GD-2011 | KP771766 | 2011 | China |  |
| 265 | 10ZQ-GD | JX192639 | 2010 | China |  |
| 266 | 10-10FUJ-2 | JQ663547 | 2010 | China |  |
| 267 | XF1129 | KT180169 | 2013 | China |  |
| 268 | NVDC-BJ1-2012 | KP771764 | 2012 | China |  |
| 269 | NVDC-BJ2-2012 | KP771763 | 2012 | China |  |
| 270 | 10-10HEB-3 | JQ663553 | 2010 | China |  |
| 271 | GD-2011 | KC527830 | 2011 | China |  |
| 272 | 09HEN1 | JF268684 | 2009 | China |  |
| 273 | NVDC-HeB2-2011 | KP771765 | 2011 | China |  |
| 274 | Shanxi-6 | KJ855518 | 2010 | China |  |
| 275 | 10-10GX-2 | JQ663559 | 2010 | China |  |
| 276 | 10-10GX-4 | JQ663561 | 2010 | China |  |
| 277 | 10QY-GD | JX215552 | 2010 | China |  |
| 278 | 10SJ-GD | JX192637 | 2010 | China |  |
| 279 | 10FS1-GD | JX192635 | 2010 | China |  |
| 280 | 10HD-GD | JX215553 | 2010 | China |  |
| 281 | 10SS-GD | JX192638 | 2010 | China |  |
| 282 | 10-10JX | JQ663540 | 2010 | China |  |
| 283 | 09HEN2 | JF268680 | 2009 | China |  |
| 284 | FS | JF796180 | 2010 | China |  |
| 285 | NVDC-BJ1-2011 | KP771778 | 2011 | China |  |
| 286 | 10-10BJ-3 | JQ663542 | 2010 | China |  |
| 287 | 10-10BJ-4 | JQ663544 | 2010 | China |  |
| 288 | 10-10FUJ-3 | JQ663548 | 2010 | China |  |
| 289 | 10-10FUJ-4 | JQ663549 | 2010 | China |  |
| 290 | NVDC-HeN-2012 | KP771771 | 2012 | China |  |
| 291 | NVDC-HuN-2011 | KP771770 | 2012 | China |  |
| 292 | 10-10GX-3 | JQ663560 | 2010 | China |  |
| 293 | 10-10BJ-2 | JQ663543 | 2010 | China |  |
| 294 | 10-10HEB-2 | JQ663552 | 2010 | China |  |
| 295 | 09JS | JF268675 | 2009 | China |  |
| 296 | BB0907 | HQ315835 | 2009 | China |  |
| 297 | JN-HS | HM016158 | 2008 | China |  |
| 298 | 10-10FUJ-5 | JQ663550 | 2010 | China |  |
| 299 | 10-10SD | JQ663555 | 2010 | China |  |
| 300 | 10-10BJ-1 | JQ663541 | 2010 | China |  |
| 301 | 10-10BJ-5 | JQ663545 | 2010 | China |  |
| 302 | 10-10GX-1 | JQ663558 | 2010 | China |  |
| 303 | DC | JF748718 | 2010 | China |  |
| 304 | 10-10GX-5 | JQ663562 | 2010 | China |  |
| 305 | 10-10FUJ-1 | JQ663546 | 2010 | China |  |
| 306 | 10-10HEB-1 | JQ663551 | 2010 | China |  |
| 307 | NVDC-YN-2011 | KP771767 | 2011 | China |  |
| 308 | 10-LW3-7 | JQ663564 | 2010 | China |  |
| 309 | 10-10LW5-1 | JQ663565 | 2010 | China |  |
| 310 | 10-LW6-6 | JQ663566 | 2010 | China |  |
| 311 | 10-LW1-13 | JQ663557 | 2010 | China |  |
| 312 | 10-LW2-6 | JQ663563 | 2010 | China |  |
| 313 | 10-LW7-1 | JQ663567 | 2010 | China |  |
| 314 | 10-LW8-1 | JQ663568 | 2010 | China |  |
| 315 | FJLIUY-2017 | MG011718 | 2017 | China |  |
| 316 | SCcd17 | MG914067 | 2017 | China |  |
| 317 | CY1-1604 | MH651736 | 2016 | China |  |
| 318 | JL580 | KR706343 | 2013 | China |  |
| 319 | SDYG1606 | KY053458 | 2016 | China |  |
| 320 | SD-1602 | MH651743 | 2016 | China |  |
| 321 | HENZMD-9 | KU950374 | 2015 | China |  |
| 322 | SC-d | MF375261 | 2015 | China |  |
| 323 | SCN17 | MH078490 | 2017 | China |  |
| 324 | FJL15 | KY412887 | 2014 | China |  |
| 325 | SDQD-1604 | MH651742 | 2016 | China |  |
| 326 | HBFL-1604 | MH651739 | 2016 | China |  |
| 327 | FJDJQ-2017 | MG011719 | 2017 | China |  |
| 328 | HNJYH-1606 | MH651740 | 2016 | China |  |
| 329 | HENAN-HEB | KJ143621 | 2012 | China |  |
| 330 | HENAN-XINX | KF611905 | 2013 | China |  |
| 331 | SDbz16-2 | MH588710 | 2016 | China |  |
| 332 | HNJYF-1606 | MH651738 | 2016 | China |  |
| 333 | HNyc15 | KT945018 | 2015 | China |  |
| 334 | SD17-38 | MH068878 | 2017 | China |  |
| 335 | SDZC-1609 | MH651747 | 2016 | China |  |
| 336 | CHsx1401 | KP861625 | 2014 | China |  |
| 337 | WUH6 | KU523367 | 2011 | China |  |
| 338 | NADC30 | MH500776 | 2017 | China |  |
| 339 | SD-A19 | MF375260 | 2015 | China |  |
| 340 | HB17A | MG844181 | 2017 | China |  |
| 341 | TJZH-1607 | MH651748 | 2016 | China |  |
| 342 | QHD3 | MH167388 | 2017 | China |  |
| 343 | LNCH-1604 | MH651741 | 2016 | China |  |
| 344 | QHD2 | MH167387 | 2017 | China |  |
| 345 | HENXX-1 | KU950372 | 2014 | China |  |
| 346 | HNjz15 | KT945017 | 2015 | China |  |
| 347 | HENXC-4 | KU950371 | 2015 | China |  |
| 348 | WUH5 | KU523366 | 2015 | China |  |
| 349 | CY2-1604 | MH651737 | 2016 | China |  |
| 350 | SD99-1606 | MH651745 | 2016 | China |  |
| 351 | SD17-36 | MH121061 | 2017 | China |  |
| 352 | SDQZ-1609 | MH651746 | 2016 | China |  |
| 353 | QHD1 | MG687491 | 2017 | China |  |
| 354 | SD53-1603 | MH651744 | 2016 | China |  |
| 355 | LNWK130 | MG913987 | 2017 | China |  |
| 356 | LNWK96 | MG860516 | 2017 | China |  |
| 357 | SD | ON254651 | 2016 | China |  |
| 358 | CH/SCZG-1/2016 | MZ747449 | 2016 | China |  |
| 359 | CH/SCMS-1/2016 | MZ747440 | 2016 | China |  |
| 360 | SD110-1608 | MK780825 | 2016 | China |  |
| 361 | SX2-1607 | MN046241 | 2016 | China |  |
| 362 | SX1-1607 | MN046240 | 2016 | China |  |
| 363 | FZ16A | KY761966 | 2016 | China |  |
| 364 | SD-R | ON254650 | 2017 | China |  |
| 365 | PRRSV2/CN/F7/2017 | OL422830 | 2017 | China |  |
| 366 | PRRSV2/CN/N42/2017 | OL422828 | 2017 | China |  |
| 367 | PRRSV2/CN/N3/2017 | OL422827 | 2017 | China |  |
| 368 | Marc-145 | MZ540774 | 2017 | China |  |
| 369 | CH/SCSN-1/2017 | MZ747445 | 2017 | China |  |
| 370 | JS1703-21 | MN547964 | 2017 | China |  |
| 371 | SDWH27-1710 | MK780824 | 2017 | China |  |
| 372 | PRRSV2/CN/110713/2018 | MT416546 | 2017 | China |  |
| 373 | PRRSV2/CN/F1004/2017 | MT416544 | 2017 | China |  |
| 374 | PRRSV2/CN/F1228/2017 | MT416543 | 2017 | China |  |
| 375 | PRRSV2/CN/X4839/2017 | MT409692 | 2017 | China |  |
| 376 | TA-12-C | MZ399801 | 2007 | China |  |
| 377 | JX07 | MN606305 | 2007 | China |  |
| 378 | GDQY1VP80 | JN387273 | 2010 | China |  |
| 379 | GDQY1VP65 | JN387272 | 2010 | China |  |
| 380 | NT0801P50 | KJ523896 | 2010 | China |  |
| 381 | JXM100 | GQ475526 | 2009 | china |  |
| 382 | JXM80 | GQ499196 | 2009 | china |  |
| 383 | JXM60 | GQ499195 | 2009 | china |  |
| 384 | JXM40 | GQ499194 | 2009 | china |  |
| 385 | JXM20 | GQ499193 | 2009 | china |  |
| 386 | NT0801P30 | KJ523895 | 2009 | China |  |
| 387 | NT0801P10 | KJ523894 | 2008 | China |  |
| 388 | GXXNF53-1805 | ON462047 | 2018 | China |  |
| 389 | CH/SCYB-1/2018 | MZ747447 | 2018 | China |  |
| 390 | CH/SCHY/2018 | MZ747439 | 2018 | China |  |
| 391 | CH/SCCD-2/2018 | MZ747437 | 2018 | China |  |
| 392 | CSR1801 | OM743305 | 2018 | China |  |
| 393 | PRRSV2/CN/L5/2018 | OM141114 | 2018 | China |  |
| 394 | PRRSV2/CN/F0/2018 | OL422832 | 2018 | China |  |
| 395 | PRRSV2/CN/Z8/2018 | OL422831 | 2018 | China |  |
| 396 | PRRSV2/CN/S5/2018 | OL422829 | 2018 | China |  |
| 397 | PRRSV2/CN/J8/2018 | OL422826 | 2018 | China |  |
| 398 | PRRSV2/CN/H4/2018 | OL422825 | 2018 | China |  |
| 399 | PRRSV2/CN/G7/2018 | OL416126 | 2018 | China |  |
| 400 | PRRSV2/CN/F5/2018 | OL416125 | 2018 | China |  |
| 401 | PRRSV2/CN/E9/2018 | OL416124 | 2018 | China |  |
| 402 | CHN-HB-2018 | MZ043753 | 2018 | China |  |
| 403 | TS01 | MT663768 | 2018 | China |  |
| 404 | HB18-41 | MT268280 | 2018 | China |  |
| 405 | KZ2018 | MN550991 | 2018 | China |  |
| 406 | JS18-3 | MN606304 | 2018 | China |  |
| 407 | SW2018001-YL | MN401750 | 2018 | China |  |
| 408 | NPUST-2789-3W-2 | MN242825 | 2018 | China |  |
| 409 | HLJZD22-1812 | MN648450 | 2018 | China |  |
| 410 | LNDZD10-1806 | MN648054 | 2018 | China |  |
| 411 | GDsc1809 | MT394497 | 2018 | China |  |
| 412 | GDxn1808 | MT394495 | 2018 | China |  |
| 413 | GDsc1808 | MT394496 | 2018 | China |  |
| 414 | GDhh1808 | MT394494 | 2018 | China |  |
| 415 | PRRSV2/CN/GDDX/2018 | MT721741 | 2018 | China |  |
| 416 | PRRSV2/CN/101805/2018 | MT416548 | 2018 | China |  |
| 417 | PRRSV2/CN/101806/2018 | MT416545 | 2018 | China |  |
| 418 | PRRSV2/CN/N9185/2018 | MT416542 | 2018 | China |  |
| 419 | PRRSV2/CN/X2984/2018 | MT416541 | 2018 | China |  |
| 420 | PRRSV2/CN/X4833/2018 | MT409691 | 2018 | China |  |
| 421 | PRRSV2/CN/X2998/2018 | MT409690 | 2018 | China |  |
| 422 | PRRSV2/CN/X4836/2018 | MT409689 | 2018 | China |  |
| 423 | PRRSV2/CN/X9830/2018 | MT409688 | 2018 | China |  |
| 424 | PRRSV2/CN/X4831/2018 | MT409687 | 2018 | China |  |
| 425 | HL85 | MN927229 | 2018 | China |  |
| 426 | HeB47 | MN927228 | 2018 | China |  |
| 427 | HeB3 | MN927227 | 2018 | China |  |
| 428 | GDDX-2018 | MT379661 | 2018 | China |  |
| 429 | GXNN1839 | MN660070 | 2018 | China |  |
| 430 | HLJ-DZD4-1805 | MN046243 | 2018 | China |  |
| 431 | LN-DB87 | MN046242 | 2018 | China |  |
| 432 | GDqy-1909 | OM949993 | 2019 | China |  |
| 433 | CH/SCYB-2/2019 | MZ747448 | 2019 | China |  |
| 434 | CH/SCYA-1/2019 | MZ747446 | 2019 | China |  |
| 435 | CH/SCMS-3/2019 | MZ747441 | 2019 | China |  |
| 436 | PRRSV2/CN/J2/2019 | OL422840 | 2019 | China |  |
| 437 | PRRSV2/CN/F2/2019 | OL422836 | 2019 | China |  |
| 438 | PRRSV2/CN/L2/2019 | OL422835 | 2019 | China |  |
| 439 | PRRSV2/CN/L1/2019 | OL422834 | 2019 | China |  |
| 440 | PRRSV2/CN/C2/2019 | OL422833 | 2019 | China |  |
| 441 | PRRSV2/CN/N4/2019 | OL422823 | 2019 | China |  |
| 442 | PRRSV2/CN/I9/2018 | OL416129 | 2019 | China |  |
| 443 | PRRSV2/CN/H1/2018 | OL416128 | 2019 | China |  |
| 444 | PRRSV2/CN/G9/2018 | OL416127 | 2019 | China |  |
| 445 | HB19-18 | MW651976 | 2019 | China |  |
| 446 | GD1909 | MT165636 | 2019 | China |  |
| 447 | SC/DJY | MT075480 | 2019 | China |  |
| 448 | JSTZ1907-714 | MN547967 | 2019 | China |  |
| 449 | JSTZ1904-664 | MN547966 | 2019 | China |  |
| 450 | HLHDZD32-1901 | MN648449 | 2019 | China |  |
| 451 | HLJZD30-1902 | MN648055 | 2019 | China |  |
| 452 | FJNP2017 | MH046842 | 2017 | China |  |
| 453 | HNRZ | MH663433 | 2017 | China |  |
| 454 | GZgy17 | MK144542 | 2017 | China |  |
| 455 | GDsf1707 | MK396376 | 2017 | China |  |
| 456 | GDsf1710 | MK396377 | 2017 | China |  |
| 457 | GDsf1711 | MK396378 | 2017 | China |  |
| 458 | XJ17-5 | MK759853 | 2017 | China |  |
| 459 | JSTZ1712-12 | MK906026 | 2017 | China |  |
| 460 | HEB-108 | MN046224 | 2017 | China |  |
| 461 | Anhui-2017-109 | MN046231 | 2017 | China |  |
| 462 | Liaoning-2017-6 | MN046233 | 2017 | China |  |
| 463 | Gansu-2017-51 | MN046235 | 2017 | China |  |
| 464 | Sichuan-2017-117 | MN046236 | 2017 | China |  |
| 465 | SD-YL1712 | MT708500 | 2017 | China |  |
| 466 | AH-PRRS20178-1 | MW853923 | 2017 | China |  |
| 467 | HM1710 | OQ924466 | 2017 | China |  |
| 468 | CH/2018/NCV-Anheal-1 | MH370474 | 2018 | China |  |
| 469 | SCya18 | MK144543 | 2018 | China |  |
| 470 | FJ0908 | MK202794 | 2018 | China |  |
| 471 | GDsf1802 | MK396379 | 2018 | China |  |
| 472 | GDsf1804 | MK396380 | 2018 | China |  |
| 473 | GDsf1806 | MK396381 | 2018 | China |  |
| 474 | GDsf1807 | MK396382 | 2018 | China |  |
| 475 | GDsf1808 | MK396383 | 2018 | China |  |
| 476 | GDsf1809 | MK396384 | 2018 | China |  |
| 477 | SWU/MS2/2018 | MK429980 | 2018 | China |  |
| 478 | SWU/MS3/2018 | MK429981 | 2018 | China |  |
| 479 | SWU/MY5/2018 | MK429982 | 2018 | China |  |
| 480 | SWU/MY6/2018 | MK429983 | 2018 | China |  |
| 481 | SWU/YB1/2018 | MK429984 | 2018 | China |  |
| 482 | SWU/YB2/2018 | MK429985 | 2018 | China |  |
| 483 | SWU/CD1/2018 | MK429986 | 2018 | China |  |
| 484 | SWU/CQ1/2018 | MK429987 | 2018 | China |  |
| 485 | CH-WH-2019-1 | MK450333 | 2018 | China |  |
| 486 | CH-YY | MK450365 | 2018 | China |  |
| 487 | HLJ-DZD1-1804 | MN046223 | 2018 | China |  |
| 488 | HeB-239 | MN046229 | 2018 | China |  |
| 489 | HLJWK108-1711 | MN046230 | 2018 | China |  |
| 490 | JS3-1805 | MN046232 | 2018 | China |  |
| 491 | HLJ-YC8 | MN046239 | 2018 | China |  |
| 492 | HN1804-2 | MN119307 | 2018 | China |  |
| 493 | JS1810-195 | MN119308 | 2018 | China |  |
| 494 | FJDJQ-2018 | MN862433 | 2018 | China |  |
| 495 | YNAN2018 | MT811822 | 2018 | China |  |
| 496 | YNCL2018 | MT811825 | 2018 | China |  |
| 497 | YNSL2018 | MT811835 | 2018 | China |  |
| 498 | YNML2018 | MT811841 | 2018 | China |  |
| 499 | HB18-36 | MW627193 | 2018 | China |  |
| 500 | HBap4/2018 | MZ579701 | 2018 | China |  |
| 501 | JSYZ1909-16 | MT780871 | 2019 | China |  |
| 502 | PRRSV2/CN/SS0/2020 | ON365556 | 2020 | China |  |
| 503 | PRRSV2/CN/H5/2020 | OL422839 | 2020 | China |  |
| 504 | PRRSV2/CN/H2/2020 | OL422838 | 2020 | China |  |
| 505 | PRRSV2/CN/F3/2020 | OL422837 | 2020 | China |  |
| 506 | PRRSV2/CN/F8/2020 | OL422824 | 2020 | China |  |
| 507 | PRRSV2/CN/L4/2020 | OL422822 | 2020 | China |  |
| 508 | GXGG202007 | OL439476 | 2020 | China |  |
| 509 | GXNN202004 | MW561594 | 2020 | China |  |
| 510 | JSYC20-05- 1 | MT746146 | 2020 | China |  |
| 511 | 2020-Acheng- 1 | MW079495 | 2020 | China |  |
| 512 | GXNN202004a | MW531679 | 2020 | China |  |
| 513 | GXNN202010 | MW561593 | 2020 | China |  |
| 514 | PRRSV-China/SCcd2020/2020 | MW803134 | 2020 | China |  |
| 515 | SXSZ-2020 | MW880772 | 2020 | China |  |
| 516 | SD-QD-2101 | MZ172971 | 2020 | China |  |
| 517 | JS2020 | MZ342900 | 2020 | China |  |
| 518 | GX20210501 | OQ204111 | 2021 | China |  |
| 519 | HN0713 | OM293962 | 2021 | China |  |
| 520 | GDHY0425 | OM293960 | 2021 | China |  |
| 521 | GDGZ0408 | OM293959 | 2021 | China |  |
| 522 | HB2104 | MZ712110 | 2021 | China |  |
| 523 | PRRSV2/CN/SS1/2021 | ON093974 | 2021 | China |  |
| 524 | FJ1805 | MZ146722 | 2021 | China |  |
| 525 | SD1805 | MZ146721 | 2021 | China |  |
| 526 | ZJqz21 | OK274266 | 2021 | China |  |
| 527 | YC-2020 | ON180781 | 2021 | China |  |
| 528 | YL-2021 | MZ169406 | 2021 | China |  |
| 529 | PRRSV2/CN/Z0/2021 | OL422844 | 2021 | China |  |
| 530 | PRRSV2/CN/Q9/2021 | OL422843 | 2021 | China |  |
| 531 | PRRSV2/CN/N0/2021 | OL422842 | 2021 | China |  |
| 532 | PRRSV2/CN/N2/2021 | OL422841 | 2021 | China |  |
| 533 | PRRSV2/CN/L3/2021 | OL416130 | 2021 | China |  |
| 534 | PRRSV2/CN/FJGD01/2021 | OL310959 | 2021 | China |  |
| 535 | GX5416 | OM202899 | 2021 | China |  |
| 536 | GX4934 | OM202898 | 2021 | China |  |
| 537 | GX4852 | OM202897 | 2021 | China |  |
| 538 | GX3251 | OM202896 | 2021 | China |  |
| 539 | GXHX20211106 | OQ459665 | 2021 | China |  |
| 540 | GXGG20210301 | OQ459663 | 2021 | China |  |
| 541 | CHbj2103 | OP734318 | 2021 | China |  |
| 542 | CHbj2101 | OP734316 | 2021 | China |  |
| 543 | CHbj2102 | OP734317 | 2021 | China |  |
| 544 | PRRSV2/CN/FJLX06/2021 | OQ357724 | 2021 | China |  |
| 545 | TZJ2451 | OQ790146 | 2022 | China |  |
| 546 | WK621 | OQ790147 | 2022 | China |  |
| 547 | TZJ2756 | OQ748875 | 2022 | China |  |
| 548 | GD2022 | OQ606399 | 2022 | China |  |
| 549 | GXBY20220301 | OQ459662 | 2022 | China |  |
| 550 | GXGL20220301 | OQ459664 | 2022 | China |  |
| 551 | GD20220303 | OQ459668 | 2022 | China |  |
| 552 | GXYN20220502 | OQ459667 | 2022 | China |  |
| 553 | GXYL20220501 | OQ459666 | 2022 | China |  |
| 554 | CH-HNPY-01/2022 | OP716076 | 2022 | China |  |
| 555 | PRRSV-CH-SDLY27-2022 | OP805381 | 2022 | China |  |
| 556 | SDwh1403 | MN642101 | 2014 | China |  |
| 557 | FS-GD-02_FoShan_Guangdong | MT036897 | 2016 | China |  |
| 558 | SDqd1501 | MN642099 | 2015 | China |  |
| 559 | SDwh1601 | MN642102 | 2016 | China |  |
| 560 | SDyt1401 | MN642105 | 2014 | China |  |
| 561 | GXNN1396-p3 | MN660067 | 2013 | China |  |
| 562 | QH- 1(sh)/2008 | KT033733 | 2008 | China |  |
| 563 | Pjx143 | EF488048 | 2007 | China |  |
| 564 | HLJ-80 | MN046222 | 2016 | China |  |
| 565 | JSWA | KY373214 | 2014 | China |  |
| 566 | 15HEN1 | KX815413 | 2015 | China |  |
| 567 | 15JX1 | KX815419 | 2015 | China |  |
| 568 | 15LN3 | KX815425 | 2015 | China |  |
| 569 | 15SC3 | KX815428 | 2015 | China |  |
| 570 | 2014-81 | MN046221 | 2014 | China |  |
| 571 | FJ1402 | KX169191 | 2014 | China |  |
| 572 | FJM4 | KY412888 | 2014 | China |  |
| 573 | Fujian-2014- 18 | MN046225 | 2014 | China |  |
| 574 | HENJY-2 | KX900392 | 2015 | China |  |
| 575 | HeNXX-2014- 12 | MN046228 | 2014 | China |  |
| 576 | HeNXX-2014-3 | MN046226 | 2014 | China |  |
| 577 | HeNXX-2014-9 | MN046227 | 2014 | China |  |
| 578 | HENXX-8 | KY041782 | 2016 | China |  |
| 579 | TJnh1501 | KX510269 | 2015 | China |  |
| 580 | SCcd16 | MF196905 | 2016 | China |  |
| 581 | 15HEB1 | KX815411 | 2015 | China |  |
| 582 | GDsg | KX621003 | 2015 | China |  |
| 583 | JX/CH/2016 | KY495780 | 2016 | China |  |
| 584 | HiNZWQ | KY373215 | 2014 | China |  |
| 585 | HNhx | KX766379 | 2016 | China |  |
| 586 | HENXX-9 | KY290748 | 2016 | China |  |
| 587 | SCnj16 | MF196906 | 2016 | China |  |
| 588 | ZJnb16-2 | MH236426 | 2016 | China |  |
| 589 | GXBB16-1 | MN026346 | 2016 | China |  |
| 590 | SDJM-1602 | MN046234 | 2016 | China |  |
| 591 | HN-1603 | MN046238 | 2016 | China |  |
| 592 | SD1612-1 | MN119304 | 2016 | China |  |
| 593 | SDwh1602 | MN642103 | 2016 | China |  |
| 594 | YNCX2016 | MT811827 | 2016 | China |  |
| 595 | YNJN2016 | MT811829 | 2016 | China |  |
| 596 | YNLQ2016 | MT811831 | 2016 | China |  |
| 597 | YNSM2016 | MT811836 | 2016 | China |  |
| 598 | GDhy-1809 | OM949992 | 2018 | China |  |
| 599 | BJ2021 | OK095299 | 2021 | China |  |
| 600 | YNPL2016 | MT811838 | 2016 | China |  |
| 601 | GXFCG20210401 | OK486522 | 2021 | China |  |
| 602 | HY21 | OL687155 | 2021 | China |  |
| 603 | GXNN20210506 | OK486524 | 2021 | China |  |
| 604 | GXQZ20210403 | OK486523 | 2021 | China |  |
| 605 | BJ20-06 | MZ047780 | 2020 | China |  |
| 606 | SDlz20-04 | MZ047781 | 2020 | China |  |
| 607 | North American | MZ160905 | 2020 | China |  |
| 608 | CH/SCMY-3/2020 | MZ747442 | 2020 | China |  |
| 609 | CH/SCNC-2/2020 | MZ747443 | 2020 | China |  |
| 610 | CH/SCPZ/2020 | MZ747444 | 2020 | China |  |
| 611 | GDYJ1224 | OM293961 | 2020 | China |  |
| 612 | TZJ226 | OP566682 | 2020 | China |  |
| 613 | TZJ637 | OP566683 | 2020 | China |  |
| 614 | CH/SCGY-2/2020 | MZ747438 | 2020 | China |  |
| 615 | HNLCL15-1903 | ON462043 | 2019 | China |  |
| 616 | HNTZJ1714-2011 | ON462044 | 2020 | China |  |
| 617 | SCCD22 | OR670493 | 2022 | China |  |
| 618 | 2023GD-4 | OR269980 | 2023 | China |  |
| 619 | BDSP-1 | OR662185 | 2023 | China |  |
| 620 | CHNMGKL1-2304 | OR753369 | 2023 | China |  |
| 621 | GD-7 | OR711915 | 2023 | China |  |
| 622 | GX-3 | OR582383 | 2023 | China |  |
| 623 | HN-NY/2023 | OR575928 | 2023 | China |  |
| 624 | PRRSV1/CN/FJFQ-1/2023 | OR260421 | 2023 | China |  |
| 625 | PRRSV1/CN/FJFQ-4/2023 | OR502390 | 2023 | China |  |
| 626 | AHBZ | KY373216 | 2014 | China |  |
| 627 | NVDC-R224-2014 | KP771783 | 2014 | China |  |
| 628 | BL2019 | OQ735301 | 2019 | China |  |
| 629 | PRRSV/S136 | OM201179 | 2019 | China |  |
| 630 | PRRSV/HB94 | OM201193 | 2019 | China |  |
| 631 | PRRSV/G101 | OM201185 | 2019 | China |  |
| 632 | HuN-ZZ | OR250810 | 2022 | China |  |
| 633 | SCABTC-202305 | OR365672 | 2022 | China |  |
| 634 | AHJ4 | PP033240 | 2022 | China |  |
| 635 | hy-2203 | OR800933 | 2022 | China |  |
| 636 | SCABTC-202302 | OQ986591 | 2022 | China |  |
| 637 | GXFS20220302 | PP625730 | 2022 | China |  |
| 638 | PRRSV2-XJ/CJ04 | OP866754 | 2022 | China |  |
| 639 | CH/SCCD-4/2020 | OL771206 | 2020 | China |  |
| 640 | GSFEI12-2023 | PP409066 | 2023 | China |  |
| 641 | GXWZ20230831 | PP824649 | 2023 | China |  |
| 642 | TZJ3005 | OR826313 | 2023 | China |  |
| 643 | WK730 | OR826314 | 2023 | China |  |
| 644 | TZJ3115 | OR826315 | 2023 | China |  |
| 645 | TZJ3116 | OR826316 | 2023 | China |  |
| 646 | SCABTC-202308 | OR365675 | 2023 | China |  |
| 647 | SCABTC-202309 | OR766560 | 2023 | China |  |
| 648 | HN2023-4 | PP065921 | 2023 | China |  |
| 649 | HN2023-11 | PP065922 | 2023 | China |  |
| 650 | GSLX2-2023 | PP409068 | 2023 | China |  |
| 651 | GDMZ0309-4 | PP065917 | 2023 | China |  |
| 652 | GDZS0706-35 | PP065919 | 2023 | China |  |
| 653 | GSTS4-2023 | PP409067 | 2023 | China |  |
| 654 | GSBY4-2023 | PP409069 | 2023 | China |  |
| 655 | SDHY-DZ037 | OP168793 | 2023 | China |  |
| 656 | GDHY1207-7 | PP065923 | 2023 | China |  |
| 657 | GDMZ0725-1 | PP065920 | 2023 | China |  |
| 658 | SCABTC-202306 | OR365673 | 2023 | China |  |
| 659 | SCABTC-202307 | OR365674 | 2023 | China |  |
| 660 | BZ-4-19 | OR115682 | 2023 | China |  |
| 661 | GDYJ0718-7 | PP065918 | 2023 | China |  |
| 662 | PRRSV/H64 | OM201192 | 2019 | China |  |
| 663 | PRRSV/S130 | OM201178 | 2019 | China |  |
| 664 | PRRSV/S145 | OM201180 | 2019 | China |  |
| 665 | PRRSV/C103 | OM201171 | 2019 | China |  |
| 666 | PRRSV/HB96 | OM201194 | 2019 | China |  |
| 667 | CH/GX/PRRSV/2475/2019 | MZ219271 | 2019 | China |  |
| 668 | CH/GX/PRRSV/2526/2019 | MZ219272 | 2019 | China |  |
| 669 | SCABTC-202304 | OQ986589 | 2022 | China |  |
| 670 | SCABTC-202303 | OQ986592 | 2022 | China |  |
| 671 | SCABTC-202301 | OQ986590 | 2022 | China |  |
| 672 | SDQD95 | OQ538073 | 2022 | China |  |
| 673 | SDYT91 | OQ538074 | 2022 | China |  |
| 674 | SDWH86 | OQ506516 | 2022 | China |  |
| 675 | XJ1904-39 | MN119309 | 2019 | China |  |
| 676 | JSTZ1810-220 | MN547965 | 2019 | China |  |
| 677 | GD-100th | GU143913 | 2009 | China |  |
