# Supplementary material for: Epidemiology and Pathogenicity Analysis Based on Partial Recombinant PRRSV Strains in China
Source: Transbound Emerg Dis. 2025 Aug 13;2025:1748117. doi: 10.1155/tbed/1748117 (PMC12367394; doi:10.1155/tbed/1748117)
Supplement: Supporting Information 2 — Table S2: Information on recombination events of four strains, detected by RPD4 software. [file 1748117.f2.docx]

| Table S2 Information on recombination events of four strains, detected by RPD4 software. | | | | | | | | | | | |
| --- | --- | --- | --- | --- | --- | --- | --- | --- | --- | --- | --- |
| Isolate | Breakpoint position in alignment | | Major parent (Similarity) | Minor parent (Similarity) | *p*-Value of the Detection Methods | | | | | | |
|  | Beginning | Ending |  |  | RDP | GENECONV | BootScan | MaxChi | Chimaera | SiScan | 3Seq |
| ZJ-2021-1 | 1743 | 5865 | JXA1 (87.5％) | NADC30 (90.9％) | 1.380×10^-62^ | 4.904×10^-34^ | 5.543×10^-56^ | 2.251×10^-31^ | 2.239×10^-34^ | 2.178×10^-47^ | 1.962×10^-09^ |
|  | 5865 | 6895 | JXA1 (90.5％) | VR2332 (93.9％) | 4.649×10^-21^ | - | 1.003×10^-19^ | 9.326×10^-14^ | 2.260×10^-14^ | 8.121×10^-11^ | 1.971×10^-11^ |
|  | 11909 | 12739 | JXA1 (92.3％) | QYYZ (89.2％) | 1.765×10^-05^ | - | - | 2.171×10^-03^ | 8.282×10^-06^ | - | 2.773×10^-06^ |
|  | 13771 | 14681 | JXA1 (91.2％) | QYYZ (89.2％) | 6.948×10^-16^ | - | 2.381×10^-13^ | 2.913×10^-08^ | 6.765×10^-10^ | 6.677×10^-07^ | 5.928×10^-13^ |
| JS-2020-1 | 616 | 2002 | NADC30 (94.1％) | JXA1 (91.6％) | 3.092×10^-90^ | 6.027×10^-19^ | 4.532×10^-87^ | 2.640×10^-28^ | 8.306×10^-30^ | 3.565×10^-27^ | 2.220×10^-15^ |
| GZ-2022-1 | 1999 | 3602 | JXA1 (93.8％) | NADC30 (90.7％) | 7.395×10^-93^ | 4.748×10^-47^ | 4.275×10^-69^ | 5.035×10^-30^ | 3.005×10^-33^ | 9.791×10^-27^ | 4.662×10^-14^ |
| SH-2020-2 | 12827 | 13420 | NADC30 (93.4％) | JXA1 (92.9％) | 1.689×10^-23^ | 2.206×10^-14^ | 1.763×10^-29^ | 2.239×10^-09^ | 1.327×10^-10^ | 1.484×10^-08^ | 2.797×10^-14^ |

Breakpoint position in PRRSV genome with reference to the VR-2332 strain.
